# Correction: A theoretical study on the on–off phosphorescence of novel Pt(ii)/Pt(iv)–bisphenylpyridinylmethane complexes

**DOI:** 10.1039/d3ra90126b

**Published:** 2024-01-08

**Authors:** Guoxun Zhu, Zhenping Chen, Huacan Song, Ao You, Zhengquan Li

**Affiliations:** a Guangdong Provincial Key Laboratory of Chemical Measurement and Emergency Test Technology, Institute of Analysis, Guangdong Academy of Sciences (China National Analytical Center, Guangzhou) Guangzhou 510070 P. R. China lzq@fenxi.com.cn; b School of Chemical Engineering and Technology, Sun Yat-sen University Zhuhai 519082 P. R. China; c School of Eco-Environmental Technology, Guangdong Industry Polytechnic 152 Xingang West Road Guangzhou 510300 P. R. China 2015100029@gdip.edu.cn

## Abstract

Correction for ‘A theoretical study on the on–off phosphorescence of novel Pt(ii)/Pt(iv)–bisphenylpyridinylmethane complexes’ by Guoxun Zhu *et al.*, *RSC Adv.*, 2022, **12**, 18238–18244, https://doi.org/10.1039/D2RA03060H

The author regrets that the funding information was incorrectly shown in the acknowledgements section of the original manuscript. The corrected funding acknowledgement is as shown below.

This work was supported by the funding of the Special Fund Projects of Guangdong Academy of Sciences for the Construction of Domestic First-class Research Institutions (2021GDASYL-20210103035), Education Commission of Guangdong Province (2020ZDZX2077), and Science and Technology Planning Project of Guangzhou (202102021119).

The Royal Society of Chemistry apologises for these errors and any consequent inconvenience to authors and readers.

## Supplementary Material

